# Marine Pharmacology in 2009–2011: Marine Compounds with Antibacterial, Antidiabetic, Antifungal, Anti-Inflammatory, Antiprotozoal, Antituberculosis, and Antiviral Activities; Affecting the Immune and Nervous Systems, and other Miscellaneous Mechanisms of Action ^†^

**DOI:** 10.3390/md11072510

**Published:** 2013-07-16

**Authors:** Alejandro M. S. Mayer, Abimael D. Rodríguez, Orazio Taglialatela-Scafati, Nobuhiro Fusetani

**Affiliations:** 1 Department of Pharmacology, Chicago College of Osteopathic Medicine, Midwestern University, 555 31st Street, Downers Grove, Illinois 60515, USA; 2 Department of Chemistry, University of Puerto Rico, San Juan, Puerto Rico 00931, USA; E-Mail: abimael.rodriguez1@upr.edu; 3 Department of Pharmacy, University of Naples “Federico II”, Via D. Montesano 49, I-80131 Napoli, Italy; E-Mail: scatagli@unina.it; 4 Fisheries and Oceans Hakodate, Hakodate 041-8611, Japan; E-Mail: anobu@fish.hokudai.ac.jp

**Keywords:** drug, marine, chemical, metabolite, natural, product, pharmacology, pharmaceutical, review, toxicology

## Abstract

The peer-reviewed marine pharmacology literature from 2009 to 2011 is presented in this review, following the format used in the 1998–2008 reviews of this series. The pharmacology of structurally-characterized compounds isolated from marine animals, algae, fungi and bacteria is discussed in a comprehensive manner. Antibacterial, antifungal, antiprotozoal, antituberculosis, and antiviral pharmacological activities were reported for 102 marine natural products. Additionally, 60 marine compounds were observed to affect the immune and nervous system as well as possess antidiabetic and anti-inflammatory effects. Finally, 68 marine metabolites were shown to interact with a variety of receptors and molecular targets, and thus will probably contribute to multiple pharmacological classes upon further mechanism of action studies. Marine pharmacology during 2009–2011 remained a global enterprise, with researchers from 35 countries, and the United States, contributing to the preclinical pharmacology of 262 marine compounds which are part of the preclinical pharmaceutical pipeline. Continued pharmacological research with marine natural products will contribute to enhance the marine pharmaceutical clinical pipeline, which in 2013 consisted of 17 marine natural products, analogs or derivatives targeting a limited number of disease categories.

## 1. Introduction

**Figure 1:**
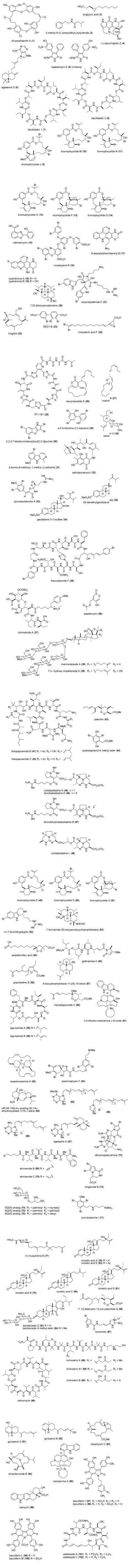
Marine pharmacology in 2009–2011: Marine compounds with antibacterial, antifungal, antiprotozoal, antituberculosis, and antiviral activities.

**Figure 2:**
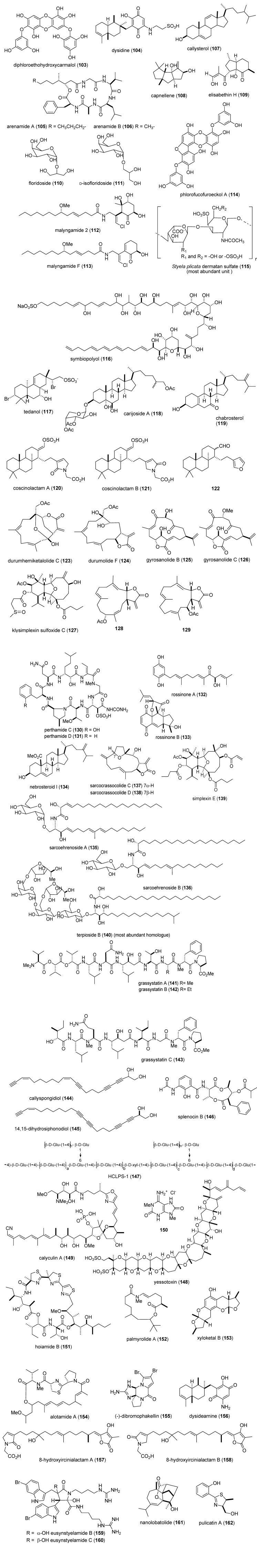
Marine pharmacology in 2009–2011:Marine compounds with antidiabetic and anti-inflammatory activity; and affecting the immune and nervous system.

**Figure 3:**
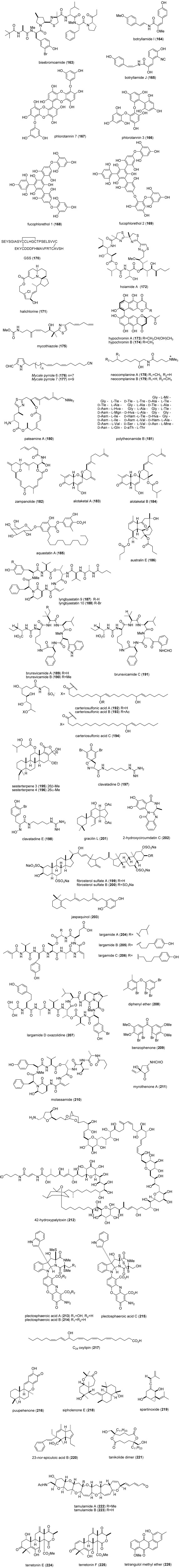
Marine pharmacology in 2009–2011: Marine compounds with miscellaneous mechanisms of action.

The current article presents a systematic review of the preclinical pharmacology of the marine natural products literature in 2009–2011, with a similar format to previous reviews [1,2,3,4,5,6,7], and which resulted from extensive searches of several databases, including Marinlit, PubMed, Current Contents^®^ and Chemical Abstracts^®^. We have limited this review to the peer-reviewed literature that reported bioactivity or pharmacology of structurally characterized marine chemicals, and have continued to use a modification of Schmitz’s chemical classification [8] to assign marine structures to six major chemical classes, namely, polyketides, terpenes, peptides, alkaloids, shikimates, and sugars. The preclinical antibacterial, antifungal, antiprotozoal, antituberculosis, and antiviral pharmacology of marine chemicals is presented in Table 1, with the corresponding structures shown in Figure 1. Marine compounds that affect the immune and nervous systems, as well as those with antidiabetic and anti-inflammatory effects are shown in Table 2, with their corresponding structures presented in Figure 2. Finally, marine compounds that have been demonstrated to affect a wide variety of cellular and molecular targets are exhibited in Table 3, and their structures presented in Figure 3. Several publications during 2009–2011 described extracts or as yet structurally uncharacterized marine compounds, and although they have been excluded from the current review, they certainly deserve further investigation because they report novel and interesting *in vitro* or *in vivo* preclinical pharmacology: *antimicrobial* and *antistaphylococcal* biofilm activity of three 5-kDa peptides isolated from coelomocyte effector cells of the sea urchin *Paracentrotus lividus* that could benefit patients with medical device-associated infections [9]; an *antibacterial* polyunsaturated fatty acid, eicosapentanoic acid, isolated from extracts of the marine diatom *Phaeodactylum tricornutum* with activity against a range of Gram-positive and Gram-negative bacteria, including multidrug-resistant *Staphyloccus aureus* [10]; potent *anticoagulant* activity of sulfated polysaccharides isolated from the Brazilian brown seaweed *Dictyota cervicornis*, which was close to that of clinically used low molecular weight heparin [11]; potent *anticoagulant* activity of a sulfated polysaccharide isolated from the Chinese green seaweed *Monostroma latissimum* by a mechanism involving thrombin inhibition in the presence of heparin cofactor II [12]; *in vitro* *antileishmanial* activity of dichloromethane extracts of a Tunisian sponge *Sarcotragus* sp., which demonstrated concomitant morphological alterations of *Leishmania major* promastigotes *in vitro* [13]; *in vivo* and *in vitro* *antifilarial* activity of the marine sponge *Haliclona exigua* extracts against adult nematode *Brugia malayi*, a parasite that may cause lymphatic filariasis [14]; significant nontoxic and anti-herpes simplex virus HSV-1 and HSV-2 activity in sulfated polysaccharide extracts isolated from four species of red and brown marine algae from New Zealand [15]; *anti-herpes* *simplex* virus HSV-1 activity in high molecular weight exopolysaccharides purified from the French marine sponge *Celtodoryx girardae* and its symbiotic bacteria [16]; *anti-inflammatory* activity of the crude extracts and fractions of the Mediterranean sponge *Spongia officinalis* in the *in vivo* rat carrageenan-induced paw edema assay [17]; *in vivo anti-inflammatory* activity in polyphenolic extracts from the red alga *Laurencia undulata* resulting in significant inhibition of asthmatic reactions [18]; *in vitro* anti-inflammatory effect in an ethanolic extract from the brown alga *Ishige okamurae* via inhibition of NF-κB transcription factor [19]; induction of *oxidative* *death* in a human glioma cell line through a caspase-9 apoptotic pathway by extracts from the marine sponge *Polymastia janeirensis* [20]; *apoptotic* activity in extracts from the marine diatom *Cocconeis scutellum* associated with activation of caspases-8 and -3 in human breast cancer lines [21]; human neutrophil *anti-elastase* activity of purified sulfated polysaccharides from the red alga *Delesseria sanguinea* [22]; high *antioxidant* activity in methanolic extracts of the Korean red alga *Polysiphonia morrowii* that protected against hydroxyl radical-induced DNA damage *in vitro* [23]; *antioxidant* activity in phenolic compounds from the marine alga *Halimeda monile* that protected against chemically induced rat liver injury *in vivo* [24]; significant *antioxidant* properties of polysaccharides from a marine fungus *Penicillium* sp. F23-2 against superoxide and hydroxyl radicals [25]; *in vitro* *antioxidant* activities of acetylated, phosphorylated and benzoylated derivatives of the marine red alga *Porphyra haitanensis* phorphyran [26]; acceleration of skin *wound healing* by amino acids isolated from the mollusc *Rapana venosa* suggesting a possible therapeutic use in skin burns [27]; *neuroprotective* effects in extracts of the South Indian green seaweed *Ulva reticulata* that inhibited both acetyl-and butyryl-cholinesterases, and was comparable to agents currently approved for Alzheimer’s disease treatment [28].

## 2. Marine Compounds with Antibacterial, Antifungal, Antiprotozoal, Antituberculosis, and Antiviral Activities

Table 1 presents the 2009–2011 preclinical pharmacological research on the antibacterial, antifungal, antiprotozoal, antituberculosis, and antiviral activities of the marine natural products (**1**–**102**) shown in Figure 1.

**Table 1:** Marine pharmacology in 2009–2011: Marine compounds with antibacterial, antifungal, antituberculosis, antiviral and other antiprotozoal activities.

Drug Class	Compound/Organism ^a^	Chemistry	Pharmacologic Activity	IC_50_^b^	MMOA ^b^	Country ^c^	References
Antibacterial	chrysophaentin A (**1**)/alga	Shikimate ^f^	Methicillin-resistant *S. aureus* inhibition	1.5 μg/mL ^+^	Inhibit GTPase activity of FtsZ	ITA, USA	[29]
Antibacterial	*H. salinus* phenethylamine (**2**)/bacterium	Shikimate ^f^	Quorum sensing inhibition	9 μg/mL	Inhibit homoserine lactone receptor binding	USA	[30]
Antibacterial	lyngbyoic acid (**3**)/cyanobacterium	Fatty acid ^d^	Quorum sensing inhibition	100 μM	Inhibit homoserine lactone receptor LasR	GBR, USA	[31]
Antibacterial	(−)-discorhabdin Z (**4**)/sponge	Alkaloid ^f^	*M. luteus* inhibition	50 μg/mL ^+^	Sortase A inhibition	S. KOR	[32]
Antibacterial	agelasine D (**5**)/sponge	Terpene ^e^	*S. epidermis* inhibition	0.09 μM ^+^	Undetermined	GBR, DEU, NDL	[33]
Antibacterial	aqabamycin E (**6**)/bacterium	Alkaloid ^f^	*P. vulgaris* inhibition	3.15 μg/mL ^+^	Undetermined	DEU	[34]
Antibacterial	bacillistatins 1–2 (**7**,**8**)/bacterium	Peptide ^f^	*S. pneumoniae* inhibition	0.5–2 μg/mL ^+^	Undetermined	USA	[35]
Antibacterial	bromophycolides (**9**–**14**)/alga	Terpene ^e^	Methicillin-resistant *S. aureus* inhibition	1.4 μM	Undetermined	FJI, USA	[36]
Antibacterial	caboxamycin (**15**)/bacterium	Alkaloid ^f^	*B. subtilis* inhibition	8 μg/mL	Undetermined	DEU	[37]
Antibacterial	crossbyanol B (**16**)/cyanobacterium	Polyketide ^d^	Methicillin-resistant *S. aureus* inhibition	2.0–3.9 μg/mL ^+^	Undetermined	USA	[38]
Antibacterial	*C. dellechiajei* alkaloids (**17**–**19**)/ascidian	Alkaloid ^f^	*E. coli* & *M. luteus* inhibition	1.1–10.5 μg/mL ^+^	Undetermined	FRA	[39]
Antibacterial	7,20-diisocyanoadociane (**20**)/sponge	Terpene ^e^	*E. coli* & *V. harvey* inhibition	1–2.5 μg/mL	Undetermined	AUS, NOR, USA	[40]
Antibacterial	eusynstyelamide F (**21**)/bryozoa	Peptide ^f^	*S. aureus* inhibition	6.25 μg/mL ^+^	Undetermined	NOR, GBR	[41]
Antibacterial	knightol (**22**)/coral	Terpene ^e^	Marine Gram + bacteria inhibition	2–8 μg ^++^	Undetermined	COL	[42]
Antibacterial	MC21-B (**23**)/bacterium	Shikimate ^f^	Methicillin-resistant *S. aureus* inhibition	1–4 μg/mL ^+^	Undetermined	JPN	[43]
Antibacterial	motualevic acid F (**24**)/sponge	Fatty acid ^e^	Methicillin-resistant *S. aureus* inhibition	1.2–3.9 μg/mL ^+^	Undetermined	USA	[44]
Antibacterial	*Nacardiopsis* thiopeptide (**25**)/bacterium	Peptide ^f^	Vancomycin-resistant *E. faecium* inhibition	1 μg/mL ^+^	Undetermined	CHE, NOR	[45]
Antibacterial	neurymenolides A–B (**26**,**27**)/alga	Polyketide ^d^	Methicillin-resistant *S. aureus* inhibition	2.1–7.8 μM	Undetermined	FJI, USA	[46]
Antibacterial	*Pseudoalteromonas* sp. metabolites (**28**,**29**)/bacterium	Polyketide ^d^	Methicillin-resistant *S. aureus* inhibition	1.9–2.2 μg/mL	Undetermined	USA	[47]
Antibacterial	pseudopterosin U (**30**)/coral	Terpene ^e^	*S. aureus* inhibition	2.9–4.5 μM	Undetermined	CAN, COL	[48]
Antibacterial	*P. vesiculosa* β-carboline (**31**)/bryozoa	Alkaloid ^f^	*B. subtilis* inhibition	2–4 μg/mL ^++^	Undetermined	NZL	[49]
Antibacterial	salinisporamycin (**32**)/bacterium	Polyketide ^d^	*S. aureus* inhibition	0.46 μg/mL ^+^	Undetermined	JPN	[50]
Antibacterial	synoxazolidinone A (**33**)/ascidian	Peptide ^f^	*C. glutamicum* inhibition	6.25 μg/mL ^+^	Undetermined	GBR, NOR	[51]
Antifungal	geodisterol sulfates ^h^ (**34**,**35**)/sponge	Steroid ^e^	*C. albicans* & *S. cerevisiae* inhibition	ND	MDR1 efflux pump inhibition	USA	[52]
Antifungal	theonellamide F (**36**)/sponge	Peptide ^f^	ND	ND	Activate 1,3-β-d-glucan synthesis	JPN	[53]
Antifungal	*P. vesiculosa* β-carboline (**31**)/bryozoa	Alkaloid ^f^	*B. subtilis* inhibition	4–5 μg/mL ^++^	Undetermined	NZL	[49]
Antifungal	citronamide A (**37**)/sponge	Peptide ^f^	*S. cerevisiae* inhibition	8 μg/mL ^+^	Undetermined	AUS	[54]
Antifungal	marmoratoside A & 17-hydroxyimpatienside A (**38**,**39**)/sea cucumber	Steroid glycoside ^e^	*C. albicans* inhibition	0.7–11 μg/mL ^+^	Undetermined	CHN	[55]
Antifungal	saadamycin(**40**)/bacterium	Polyketide ^d^	*C. albicans*, *Aspergillus* & *Cryptococcus* inhibition	1–5.2 μg/mL ^+^	Undetermined	EGY	[56]
Antifungal	theopapuamides B & C (**41**,**42**)/sponge	Peptide ^f^	*C. albicans* inhibition	1–5 μg/disk ^++^	Undetermined	ITA,USA	[57]
Antimalarial	plakortin (**43**)/sponge	Polyketide ^d^	*P. falciparum* D10 & W2 strain inhibition	ND	Toxic carbon-radical	ITA	[58]
Antimalarial	homogentisic acid (**44**)/sponge	Shikimate ^f^	*P. falciparum* FcB1 strain inhibition	12 μM	Pfnek-1 enzyme inhibition	CHE, NCL, FRA, UK	[59]
Antimalarial	batzelladine alkaloids (**45**–**48**)/sponge	Alkaloid ^f^	*P. falciparum* FcB1 strain inhibition	0.2–0.9 μM	Undetermined	COL, FRA	[60]
Antimalarial	bromophycolides (**9**–**14**)/alga	Terpene ^e^	*P. falciparum* 3D7 strain inhibition	0.5–2.9 μM	Undetermined	FJI, USA	[36]
Antimalarial	Bromophycolides R, S, U) (**49**–**51**)/alga	Terpene ^e^	*P. falciparum* 3D7 strain inhibition	0.9–2.1 μM	Undetermined	FJI, USA	[61]
Antimalarial	(+)-7-bromotrypargine (**52**)/sponge	Alkaloid ^f^	*P. falciparum* Dd2 & 3D7 strain inhibition	3.5–5.4 μM	Undetermined	AUS	[62]
Antimalarial	*C. hooperi* diterpene (**53**)/sponge	Terpene ^e^	*P. falciparum* F85, D6, W2 strain inhibition	4.3–4.7 ng/mL0.5 μg/mL	Undetermined	AUS, NOR, USA	[40,63]
Antimalarial	epiplakinidioic acid (**54**)/sponge	Polyketide ^d^	*P. falciparum* W2 strain inhibition	0.3 μg/mL	Undetermined	USA	[64]
Antimalarial	gallinamide A (**55**)/bacterium	Peptide ^f^	*P. falciparum* W2 strain inhibition	8.4 μM	Undetermined	PAN, USA	[65]
Antimalarial	gracilioether B (**56**)/sponge	Polyketide ^d^	*P. falciparum* ItG strain inhibition	0.5 μg/mL	Undetermined	JPN, NLD	[66]
Antimalarial	8-isocyanoamphilecta-11(20), 15-diene (**57**)/sponge	Terpene ^e^	*P. falciparum* K1 strain inhibition	0.94 μM	Undetermined	THAI	[67]
Antimalarial	lagunamides A & B (**58**,**59**)/bacterium	Peptide ^f^	*P. falciparum* NF54 strain inhibition	0.19–0.91 μM	Undetermined	CHE, NZL,SGP	[68]
Antimalarial	manadoperoxide C (**60**)/sponge	Polyketide ^d^	*P. falciparum* W2 strain inhibition	2.33 μM	Undetermined	ITA	[69]
Antimalarial	3,4-dihydro-manzamine J *N*-oxide (**61**)/sponge	Alkaloid ^f^	*P. falciparum* K1 strain inhibition	0.58 μg/mL	Undetermined	AUS, JPN	[70]
Antimalarial	neopetrosiamine A (**62**)/sponge	Alkaloid ^f^	*P. falciparum* inhibition	2.3 μM	Undetermined	USA	[71]
Antimalarial	psammaplysin F(**63**)/sponge	Peptide ^f^	*P. falciparum* 3D7 strain inhibition	0.87 μM	Undetermined	AUS	[72]
Antiprotozoal	*C. cervicornis* diterpene (**64**)/alga	Terpene ^e^	*L amazonensis* inhibition	2–12 μg/mL	Mitochondrial swelling & damage	BRA	[73]
Antiprotozoal	agelasine analogs (**65**–**68**)/synthetic	Terpene ^e^	*L. infantum*, *T. brucei brucei* & *T. cruzi* inhibition	0.093–0.43 μg/mL	Undetermined	BEL, NOR	[74]
Antiprotozoal	almiramides B & C (**69**,**70**)/bacterium	Peptide ^f^	*L. donovani* inhibition	1.9–2.4 μM	Undetermined	PAN, USA	[75]
Antiprotozoal	convolutamine I (**71**)/bryozoan	Alkaloid ^f^	*T. brucei brucei* inhibition	1.1 μM	Undetermined	AUS	[76]
Antiprotozoal	longamide B & dibromopalau’ amine (**72**,**73**)/sponge	Alkaloid ^f^	*L. donovani* & *T. brucei rhodesiense* inhibition	0.5–3.8 μg/mL	Undetermined	CHE, GBR, ITA	[77]
Antiprotozoal	*L. variegata* SQDG’s (**74**–**76**)/alga	Glycolipid	*E. histolytica* & *T. vaginalis* inhibition	3.9–8.0 μg/mL	Undetermined	MEX	[78]
Antiprotozoal	3,4-dihydro-manzamine J *N*-oxide (**61**)/sponge	Alkaloid ^f^	*T. brucei brucei* inhibition	0.27 μg/mL	Undetermined	AUS, JPN	[70]
Antiprotozoal	(+)-muqubilone B (**77**)/sponge	Terpene ^e^	*T. brucei brucei* inhibition	2 µg/mL	Undetermined	USA	[79]
Antiprotozoal	norselic acids A–E (**78**–**82**)/sponge	Steroid ^f^	*Leishmania* sp. inhibition	2–3.6 µM	Undetermined	USA	[80]
Antiprotozoal	pandaroside G & methyl ester (**83**,**84**)/sponge	Steroid glycoside ^e^	*L. donovani* & *T. brucei rhodesiense* inhibition	0.04–1.3 µM	Undetermined	CUB, CHE, FRA, GBR	[81]
Antiprotozoal	*Plakortis* sp. polyketide (**85**)/sponge	Polyketide ^d^	*T. brucei brucei* inhibition	0.049 µM	Undetermined	AUS	[82]
Antiprotozoal	valinomycin (**86**)/bacterium	Peptide ^f^	*L. major* & *T. brucei brucei* inhibition	0.0032–0.11 μM	Undetermined	GER,USA	[83]
Antituberculosis	hymenidin (**87**)/sponge	Alkaloid ^f^	*M. tuberculosis* H37Rvinhibition	6.1 μg/mL ^+^	Undetermined	USA	[84]
Antituberculosis	trichoderins A, A1, B (**88**–**90**)/fungus	Peptide ^f^	*M. tuberculosis* H37Rv strain inhibition	0.12–2 μg/mL ^+^	Undetermined	JPN, ZAF, USA	[85]
Antituberculosis	neopetrosiamine A (**62**)/sponge	Alkaloid ^f^	*M. tuberculosis* H37Rvinhibition	7.5 μg/mL ^+^	Undetermined	USA	[71]
Antiviral	gyrosanols A & B (**91**,**92**)/soft coral	Terpene ^e^	Human cytomegalovirus inhibition	2.6–3.7 μM	Undetermined	TWN	[86]
Antiviral	lobophynin C & ehrenberoxide B (**93**,**94**)/coral	Terpene ^e^	Human cytomegalovirus inhibition	4.7–5.8 μM	Undetermined	TWN	[87]
Antiviral	manzamine A (**95**)/sponge	Alkaloid ^f^	Human herpes simplex virus-1 inhibition	1 μM *	Early ICPO gene transcription inhibited	USA	[88]
Antiviral	xiamycin (**96**)/bacterium	Alkaloid ^f^	Inhibition of HIV-1 infection	7.2 μg/mL *	Selective inhibition of CCR5 tropic HIV	DEU, CHN	[89]
Antiviral	baculiferins (**97**–**100**)/sponge	Alkaloid ^f^	Inhibition of HIV-1 IIIB replication	0.2–7.0 μM	Binding to Vif, APOBEC3G & gp41	DEU, CHN	[90]
Antiviral	celebesides A & C (**101**,**102**)/sponge	Peptide ^f^	Inhibition of HIV-1 infectivity assay	1.9 μg/mL	Undetermined	ITA, USA	[57]

^a^ **Organism**, *Kingdom Animalia*: ascidian (Phylum Chordata), bryozoa (Phylum Bryozoa), coral (Phylum Cnidaria), sea cucumber (Phylum Echinodermata), sponge (Phylum Porifera); *Kingdom Monera*: bacterium (Phylum Cyanobacteria); *Kingdom Fungi*: fungus; *Kingdom Plantae*: alga; ^b^ **IC_50_**: concentration of a compound required for 50% inhibition *in vitro*, *: estimated IC_50_; ND: not determined; ^+^MIC: minimum inhibitory concentration; ^++^MID: minimum inhibitory concentration per disk; ^b^ **MMOA**: molecular mechanism of action; ^c^ **Country**: AUS: Australia; BEL: Belgium; BRA: Brazil; CAN: Canada; CHE: Switzerland; CHN: China; COL: Colombia; CUB: Cuba; DEU: Germany; EGY: Egypt; FJI: Fiji; FRA: France; GBR: United Kingdom; ITA: Italy; JPN: Japan; MEX: Mexico; NCL: New Caledonia; NLD: The Netherlands; NOR: Norway; NZL: New Zealand; PAN: Panama; SGP: Singapore; ZAF: S. Africa; S. KOR: South Korea; THAI: Thailand; TWN: Taiwan; UK: United Kingdom; **Chemistry:** ^d^ Polyketide; ^e^ Terpene; ^f^ Nitrogen-containing compound; **^g^** Polysaccharide, modified as in the text; ^h^ Named as sulfites in the original paper.

### 2.1. Antibacterial Activity

During 2009–2011, 35 studies reported *antibacterial* marine natural products isolated from a diverse group of marine bacteria, ascidians, bryozoans, sponges, soft corals and algae, a persistent effort on which we have reported previously [7], and which continues to contribute to the global health challenge posed by drug-resistant bacteria.

Only four papers reported molecular mechanism of action studies with marine antimicrobial compounds. Plaza and colleagues investigated bisdiarylbutene macrocycle chrysophaentin A (**1**) from the chrysophyte alga *Chrysophaeum taylori* that potently inhibited Gram positive methicillin-resistant *Staphylococus aureus* (minimum inhibitory concentration [MIC]_50_ = 1.5 μg/mL) and vancomycin-resistant *Enterococcus faecium* (MIC_50_ = 2.9 μg/mL) by binding and inhibiting GTPase activity of the essential bacterial cell division protein FtsZ [29]. Two studies contributed to the ongoing search of quorum sensing antagonists as potentially novel antimicrobial drugs: Teasdale and colleagues extended the pharmacology of two previously described phenethylamide metabolites isolated from a marine Gram positive *Halobacillus salinus* strain [30]. One of these compounds, 3-methyl-*N*-(2′-phenylethyl)-butyramide (**2**) interfered with quorum sensing-regulated activities (e.g., bioluminescence inhibition IC_50_ = 9 μg/mL) in several Gram negative species. Kwan and colleagues isolated a small cyclopropane-containing fatty acid, lyngbyoic acid (**3**) from the marine cyanobacterium *Lyngbya* cf. *majuscula* [31] that affected both quorum sensing pathways (acylhomoserine lactone receptor LAsR (IC_50_ = 100 µM) as well as gene expression in *Pseudomonas aeruginosa*. Jeon and colleagues extended the pharmacology of the pyrroloiminoquinone alkaloids of the discorhabdin class isolated from the Korean marine sponge *Sceptrella* sp. [32]. A new alkaloid (−)-discorhabdin Z (**4**), possessing a unique hemiaminal group, inhibited sortase A (IC_50_ = 6.5 μM), a bacterial transpeptidase that has been shown to covalently attach proteins to the bacterial cell wall and has become an important antimicrobial target.

As shown in Table 1 and Figure 1, several marine chemicals, some of them novel, were reported to exhibit antibacterial activity with MICs < 10 μg/mL or 10 μM against several bacterial strains, although no mechanism of action studies were reported for these compounds: a new alkaloid agelasine D (**5**) isolated from the marine sponge *Agelas nakamurai* collected in Bali, Indonesia [33]; the maleimide mixture aqabamycin E (**6**) discovered in a marine *Vibrio* sp. growing on the surface of the Red Sea soft coral *Sinularia polydactyla* [34]; two new cyclodepsipeptides, bacillistatins 1 and 2 (**7**,**8**) purified from cultures of the Chilean bacterium *Bacillus silvestris* obtained from a crab [35]; new diterpene–benzoate macrolides bromophycolides J–Q (**9**–**14**) from the Fijian red alga *Callophycus serratus* [36]; a novel benzoxazole antibiotic caboxamycin (**15**) produced by the deep-sea *Streptomyces* sp. NTK 937 isolated in the Canary Basin [37]; a brominated polyphenyl ether crossbyanol B (**16**) isolated from the Hawai’ian marine cyanobacterium *Leptolyngbya crossbyana* [38]; three novel pyridoacridine alkaloids (**17**–**19**) characterized from the Mediterranean ascidian *Cystodytes dellechiajei* [39]; the previously-described Fijian *Cymbastela hooperi* diterpene isonitrile (**20**) recently re-evaluated [40]; a novel alkaloid eusynstyelamide F (**21**), isolated from an Arctic bryozoan *Tegella* cf. *spitzbergensis* [41]; a novel cembranoid diterpene, knightol (**22**), found in the Colombian gorgonian octocoral *Eunicea knighti* [42]; the novel antibiotic MC21-B (**23**) produced by the marine bacterium *Pseudoalteromonas phenolica* O-BC-30T [43]; a novel long-chain 2*H*-azirine 2-carboxylic acid, motualevic acid F (**24**), described from a Fijian marine sponge *Siliquariaspongia* sp. [44]; the thiopeptide TP-1161 (**25**), identified in a Norwegian marine-sediment derived Gram positive *Nocardiopsis* sp. bacterium [45]; two novel α-pyrone macrolides neurymenolides A and B (**26**, **27**) isolated from the Fijian red alga *Neurymenia fraxinifolia* [46]; two polybrominated metabolites (**28**, **29**) from a Hawai’ian marine bacterium *Pseudoalteromonas* sp. found on the surface of a nudibranch [47]; pseudopterosin U (**30**) discovered in the Caribbean octocoral *Pseudopterogorgia elisabethae* [48]; 5-bromo-8-methoxy-1-methyl-β-carboline (**31**), an alkaloid isolated from the New Zealand marine bryozoan *Pterocella vesiculosa* [49]; a new rifamycin antibiotic, salinisporamycin (**32**), purified from a culture of the Micronesian marine actinomycete *Salinispora arenicora* YM23-082 [50]; a novel bioactive alkaloid, synoxazolidinone A (**33**), discovered in the sub-Arctic Norwegian ascidian *Synoicum pulmonaria* [51].

Furthermore, during 2009–2011, several marine natural products were noted to have MIC or IC_50_ ranging from 10 to 50 μg/mL, or 10–50 μM, respectively, and thus because of their lower antibacterial potency were *excluded* from Table 1 and Figure 1: a novel casbane diterpenoid 10-hydroxydepressin (MIC = 17 μg/mL) from the Hainan soft coral *Sinularia depressa* [91]; a novel guai-2-en-10α-methyl methanoate from the marine alga *Ulva fasciata* (MIC = 25–30 μg/mL) [92]; a novel gymnochrome F (MIC = 12.5 μg/mL) from the deep-water crinoid *Holopus* *rangii* [93]; several novel fatty acids, ieodomycins (MIC = 32–64 μg/mL), from a marine *Bacillus* sp. [94]; a novel sulfated sesterterpene alkaloid 19-oxofasciospongine A from a marine sponge *Fasciospongia* sp. (MIC = 20 μg/disk) [95]; a novel phenalenone derivative (MIC = 24 μM) from the marine-derived fungus *Coniothyrium cereale* [96]; a novel 4,4′-oxybis[3-phenylpropionic acid] from the marine bacterium *Bacillus licheniformis* (IC_50_ = 50 μg/disk) [97]; a novel sargafuran (MIC = 15 μg/mL) from the marine brown alga *Sargassum macrocarpum* [98]; a novel tetracyclic brominated diterpene (MIC = 32 μg/mL) from the red alga *Sphaerococcus coronopifolius* [99]; and a new bromotyrosine alkaloid tyrokeradine B (MIC = 25 μg/mL) from a Verongid marine sponge [100].

Finally, during 2009–2011, several publications described novel marine antimicrobial peptides: centrocins 1 and 2, two novel dimeric peptides (MIC = 1.3–2.5 μM) from the Norwegian green sea urchin *Strongylocentrotus droebachiensis* [101]; halocyntin and papillosin, two new peptides (MIC = 0.75–25 μM) isolated from hemocytes of the Mediterranean ascidian *Halocynthia papillosa* [102]; and hyastatin, a glycine-rich multi-domain peptide (MIC = 0.4–12.5 μM) from hemocytes of the Norwegian spider crab *Hyas araneus* [103].

### 2.2. Antifungal Activity

Ten studies during 2009–2011 reported on the *antifungal* activity of several novel marine natural products isolated from marine bacteria, sponges and bryozoa, a slight decrease from our last review [7], and previous reviews of this series.

As shown in Table 1, only two reports described antifungal marine chemicals with novel mechanisms of action. DiGirolamo and colleagues identified two new sulfated sterols, geodisterol-3-*O*-sulfate (**34**) and 29-demethylgeodisterol-3-*O*-sulfate (**35**), in a marine sponge *Topsentia* sp. [52], which enhanced the activity of the clinically used triazole antifungal agent fluconazole by reversing efflux pump-mediated fluconazole resistance in *Candida albicans*. Nishimura and colleagues extended the pharmacology of the bicyclic antifungal dodecapeptide theonellamide F (**36**) previously isolated from a sponge *Theonella* sp. [53]. Chemical-genomic profiling analysis together with detailed subcellular localization studies determined that the antifungal theonellamides represent a new class of sterol-binding molecules that induce membrane damage and activate Rho1-mediated 1,3-β-d-glucan synthesis. 

Furthermore, as shown in Table 1 and Figure 1 several marine natural products showed significant antifungal activity (*i.e*., MICs that were either less than 10 μg/mL, 10 μM, or 10 μg/disk), although no mechanism of action studies were reported in the published articles: the antibacterial alkaloid 5-bromo-8-methoxy-1-methyl-β-carboline (**31**) isolated from the New Zealand marine bryozoan *Pterocella vesiculosa* [49]; a novel linear tetrapeptide citronamide A (**37**) from the Australian sponge *Citronia astra* [54]; two holostan-type triterpenoid glycosides, marmoratoside A (**38**) and 17α-hydroxy impatienside A (**39**), from the Chinese sea cucumber *Bohadschia marmorata* [55]; the polyketide saadamycin (**40**) from an endophytic *Streptomyces* sp. strain Hedaya 48 isolated from the Egyptian sponge *Aplysina fistularis* [56], and two novel depsipeptides, theopapuamides B & C (**41**,**42**), isolated from the Indonesian marine sponge *Siliquariaspongia mirabilis* [57]. Future mechanism of action studies with these potent compounds will hopefully characterize their molecular pharmacology.

Finally, several novel structurally-characterized marine molecules demonstrated MICs or IC_50_s greater than 10 μg/mL or 10 μM, and therefore, because of the reported weaker antifungal activity, have been *excluded* from Table 1 and Figure 1: the maleimide mixture aqabamycin E (**6**) (MIC = 50 μg/mL) [34]; the novel antibacterial alkaloid synoxazolidinone A (**33**) (MIC = 12.5 μg/mL) [51], and the new bromotyrosine tyrokeradine B (MIC = 12.5 μg/mL) [100].

These marine compounds may provide novel pharmacological leads thus contributing to the global search for clinically useful antifungal agents.

### 2.3. Antiprotozoal and Antituberculosis Activity

As shown in Table 1, during 2009–2011 thirty two studies contributed to novel findings on the *antiprotozoal and antituberculosis* pharmacology of structurally characterized marine natural products, a considerable increase from previous 1998–2008 reviews [7].

Malaria, which is caused by protozoa from the genus *Plasmodium* (*P. falciparum*, *P. ovale*, *P. vivax* and *P. malariae*), affects millions of people worldwide. Contributing to the global search for novel antimalarial drugs, and as presented in Table 1, twenty six novel marine molecules were shown during 2009–2011 to possess *antimalarial activity*, although mechanism of action studies were reported for only two compounds. Taglialatela-Scafati and colleagues extended the molecular pharmacology of plakortin (**43**), isolated from the Caribbean marine sponge *Plakortis simplex*, which potently inhibited CQ-resistant strains of *Plasmodium falciparum* [58]. Plakortin was observed to give rise to toxic carbon radicals which were ultimately “responsible for subsequent reactions leading to *Plasmodium* death”. Lebouvier and colleagues reported that the homogentisic acid derivative **44** from a Vanuatu marine sponge *Pseudoceratina* sp. was moderately active *in vitro* against FcB1 *P. falciparum* strain, while concomitantly inhibiting the specific protein kinase pfnek-1 of the parasite (IC_50_ = 1.8 μM). Thus, compound **44** “could serve as a model for the development of new pfnek-1 inhibitors” [59].

Potent (<2 µM) to moderate (>2–10 µM) *antimalarial* activity was reported for 24 marine natural products. Laville and colleagues isolated several novel guanidine batzelladine alkaloids (**45**–**48**) from the Caribbean marine sponge *Monanchora arbuscula*, which showed potent antimalarial activity against the human malaria parasite *Plasmodium falciparum* strain FcB1 (IC_50_ = 0.2–0.9 μM) [60]. Lane and colleagues characterized new diterpene–benzoate macrolides bromophycolides J, M, N, O, P and Q (**9**–**14**) from the Fijian red alga *Callophycus serratus* with potent antimalarial activity against *P. falciparum* (IC_50_ = 0.5–2.9 μM) [36]. Furthermore, Lin and colleagues isolated bromophycolides R, S and U (**49**–**51**) from the same Fijian red alga *Callophycus serratus* with potent antimalarial activity (IC_50_ = 0.9–2.1 μM) against *P. falciparum* [61]. Davis and colleagues identified a novel β-carboline alkaloid, (+)-7-bromotrypargine (**52**), from an Australian marine sponge *Ancorina* sp. with moderate antimalarial activity (IC_50_ = 3.5–5.4 μM) against both chloroquine resistant (Dd2) and chloroquine-sensistive (3D7) *P. falciparum* strains [62]. Wright and colleagues reported a diterpene formamide (**53**) from the tropical marine sponge *Cyambastela hooperi* that had moderate to potent activity (IC_50_ = 4.3 ng/mL and 0.5 μg/mL) against *P. falcip*arum strains FCR3F86, W2 and D6 [40,63]. Jiménez-Romero and colleagues contributed a novel five-membered-ring polyketide endoperoxide epiplakinidioic acid (**54**) from the Puerto Rican sponge *Plakortis halichondrioides* that was potent (IC_50_ = 0.3 μg/mL) against the W2 chloroquinone-resistant strain of *P. falciparum* [64]. Linington and colleagues purified a novel linear peptide, gallinamide A (**55**), from a cyanobacterium *Schizothrix* sp. with moderate antimalarial activity (IC_50_ = 8.4 μM) against chloroquine-resistant *P. falciparum*, yet with a structure that might become an “attractive foundation for further SAR investigations” [65]. Ueoka and colleagues investigated a new polyketide, gracilioether B (**56**), from the Japanese marine sponge *Agelas gracilis* that was potent (IC_50_ = 0.5 µg/mL) against *P. falciparum* strain ItG [66]. Wattanapiromsakul and colleagues isolated a new isocyanoditerpene, 8-isocyanoamphilecta-11 (**20**), 15-diene (**57**), from a Thai sponge *Ciocalapata* sp. with potent antimalarial activity (IC_50_ = 0.98 μM) against *P. falciparum* chloroquine-resistant strain K1 [67]. Tripathi and colleagues found two new cyclic depsipeptides, lagunamides A (**58**) and B (**59**), from the Singaporean marine cyanobacterium *Lyngbya majuscula* with potent antimalarial activity (IC_50_ = 0.19–0.91 μM) against the drug-sensitive NF54 *P. falciparum* strain [68]. Fattorusso and colleagues discovered that a new endoperoxyketal, manadoperoxide C (**60**), from the Indonesian sponge *Plakortis* cf. *simplex*, had moderate *in vitro* antiplasmodial activity (IC_50_ = 2.3 μM) against the W2 chloroquinone-resistant strain of *P. falciparum* as well as provided further insight into “the structure activity relationships of simple 1,2-dioxane antimalarials” [69]. Yamada and colleagues identified a novel alkaloid 3,4-dihydro-manzamine J *N*-oxide (**61**) from an Okinawan marine sponge *Amphimedon* sp. with potent *in vitro* antiplasmodial activity (IC_50_ = 0.58 μg/mL) against the K1 strain of *P. falciparum* [70]. Wei and colleagues extracted a new tetracyclic bis-piperidine alkaloid, neopetrosiamine A (**62**), from the Puerto Rican marine sponge *Neopetrosia proxima* with moderate antiplasmodial activity (IC_50_ = 2.3 μM) against *P. falciparum* and low concomitant cytotoxicity to Vero cells [71]. Yang and colleagues extended the pharmacology of the known bromotyrosine alkaloid psammaplysin F (**63**) from an Australian marine sponge *Hyattella* sp. by reporting potent inhibition (IC_50_ = 0.87–1.4 μM) of *P. falciparum* 3D7 and Dd2 strains [72].

Eighteen marine compounds were reported to possess *activity against other protozoa* thus contributing to the ongoing global search for novel agents for the so-called neglected diseases, namely leishmaniasis (caused by several species of the genus *Leishmania*), amebiasis, trichomoniasis, African sleeping sickness (caused by *Trypanosoma brucei rhodesiense* and *Trypanosoma brucei gambiense*) and American sleeping sickness or Chagas disease (caused by *Trypanosoma cruzi*). Dos Santos and colleagues reported that a 4-acetoxydolastane diterpene (**64**) isolated from the Brazilian brown alga *Canistrocarpus cervicornis* [73] dose-dependently inhibited promastigote, axenic amastigote and intracellular amastigote forms of *Leishmania amazonensis* (IC_50_ = 2.0, 12.0 and 4.0 μg/mL, respectively) by extensive mitochondrial damage and lipid peroxidation.

As shown in Table 1 and Figure 1, several marine natural products were characterized to exhibit antiprotozoal activity, although the mechanism of action of these compounds remains undetermined. Vik and colleagues completed a comprehensive screening of agelasine terpene analogs from a marine sponge *Agelas* sp. and reported that four agelasine analogs (**65**–**68**) potently inhibited *Leishmania infantum*, *T. brucei brucei* and *T. cruzi* (IC_50_ = 0.093–0.11; 0.11–0.23; 0.11–0.43 μg/mL, respectively) with low concomitant cytotoxicity, suggesting they could become novel leads for design of “potent and selective drugs” [74]. Sanchez and colleagues found two novel linear lipopeptides, almiramides B and C (**69**,**70**), from the marine cyanobacterium *Lyngbya majuscula* that inhibited the protozoan parasite *Leishmania donovani* (IC_50_ = 1.9–2.4 μM) with minimal cytotoxicity towards Vero cells (IC_50_ = 33.1–52.3 μM) [75]. Davis and colleagues investigated a new brominated alkaloid, convolutamine I (**71**), from the bryozoan *Amathia tortusa* that was highly active towards the parasite *Trypanosoma brucei brucei* (IC_50_ = 1.1 μM), yet presenting low cytotoxicity in human embryonic kidney cells (IC_50_ = 22.0 μM) [76]. Scala and colleagues completed the first antiprotozoal screening of marine bromopyrrole alkaloids isolated from the marine sponges *Axinella* *verrucosa* and *Agelas* *dispar* and determined that longamide B (**72**) and dibromopalau’amine (**73**) inhibited *Trypanosoma brucei rhodesiense* (IC_50_ = 1.53 & 0.46 μg/mL, respectively), and *Leishmania donovani* (IC_50_ = 3.85 & 1.09 μg/mL, respectively), with low cytotoxicity [77]. Cantillo-Ciau and colleagues isolated two known and one novel sulfoquinovosyl diacylglycerols (**74**–**76**) from the Mexican tropical brown alga *Lobophora* *variegata* with high antiprotozoal activity against *E. histolytica* (IC_50_ = 3.9 μg/mL) and moderate activity towards *Trichomonas vaginalis* trophozoites (IC_50_ = 8.0 μg/mL), and with a good selectivity index (SI > 10) [78]. Yamada and colleagues characterized a novel alkaloid 3,4-dihydro-manzamine J *N*-oxide (**61**) from an Okinawan marine sponge *Amphimedon* sp. which displayed moderate *in vitro* activity against *Trypanosoma brucei brucei* (IC_50_ = 0.27 μg/mL) [70]. Rubio and colleagues evaluated several known peroxiterpenes as well as a novel (+)-muqubilone B (**77**) from the Papua New Guinea marine sponge *Diacarnus bismarckensis* shown to inhibit *Trypanosoma brucei brucei* (IC_50_ = 2 μg/mL), concluding that these marine chemicals could become “therapeutic leads” against *T. brucei* [79]. Ma and colleagues identified novel highly oxidized steroids norselic acids A–E (**78**–**82**) isolated from an Antarctic marine sponge *Crella* sp. which moderately inhibited *Leishmania* sp. (IC_50_ = 2.0–3.6 μM) [80]. Regalado and colleagues reinvestigated the Caribbean sponge *Pandaros acanthifolium* and discovered that among several new steroidal glycosides, both pandaroside G (**83**) and pandaroside G methyl ester (**84**) potently inhibited the growth of *Trypanosoma brucei rhodesiense* (IC_50_ = 0.8 and 0.038 μM, respectively), and *Leishmania donovani* (IC_50_ = 1.3 and 0.051 μM, respectively) but with rather high concomitant cytotoxicity [81]. Feng and colleagues isolated a novel cyclic polyketide peroxide, 11,12-didehydro-13-oxo-plakortide Q (**85**), from an Australian marine sponge *Plakortis* sp. with significant activity (IC_50_ = 0.049 μM) against *Trypanosoma brucei brucei* [82]. Pimentel-Elardo and colleagues reported the isolation of the known cyclic depsipeptide valinomycin (**86**) from marine *Streptomyces* sp. strains associated with several Croatian marine sponges, and observed significant activity against both *Trypanosoma brucei brucei* (IC_50_ = 0.0032 μM) and *Leishmania major* (IC_50_ < 0.11 μM) [83].

As shown in Table 1, four novel marine natural products contributed to the global search for novel *antituberculosis* agents, a decrease from our previous reviews [7].

Vicente and colleagues extended the pharmacology of the known compound hymenidin (**87**) from the Puerto Rican marine sponge *Prosuberites laughlini* by demonstrating its antimycobacterial activity (MIC = 6.1 μg/mL) against *M. tuberculosis* H_37_Rv [84]. Pruksakorn and colleagues isolated three new aminolipopeptides, trichoderins A (**88**), A1 (**89**) and B (**90**) from a marine sponge-derived fungus *Trichoderma* sp. that potently inhibited (MIC = 0.02–2.0 μg/mL) the *M. tuberculosis* strain H37Rv under both aerobic and dormancy-inducing conditions [85]. Wei and colleagues isolated tetracyclic bis-piperidine alkaloid neopetrosiamine A (**62**) from the Puerto Rican marine sponge *Neopetrosia proxima* which inhibited growth (MIC = 7.5 μg/mL) of the pathogenic strain *Mycobacterium* *tuberculosis* H_37_Rv [71]. Although all of these studies demonstrate that marine alkaloids and peptides may potentially become novel antituberculosis leads, further studies are required to determine the molecular pharmacology of these compounds.

### 2.4. Antiviral Activity

As shown in Table 1, three reports were published during 2009–2011 on the *antiviral* pharmacology of novel marine natural products against human cytomegalovirus and herpes simplex virus. Cheng and colleagues purified two new diterpenoids, gyrosanols A and B (**91**,**92**), and two novel cembranoids, lobophynin C and ehrenberoxide B (**93**,**94**), from the Taiwanese soft corals *Sinularia capillosa* and *Sarcophyton ehrenbergi*, respectively, which inhibited the herpes virus-5 or cytomegalovirus (HCMV) (IC_50_ = 2.6–5.8 μM), an interesting preclinical contribution because HCMV infections may be life-threatening in immunocompromised patients [86,87]. Palem and colleagues extended the pharmacology of the known β-carboline alkaloid manzamine A (**95**), isolated from an Indo-Pacific sponge *Acanthostrongylophora* sp., by demonstrating the compound inhibited HSV-1 infection (apparent IC_50_ = 1 μM) in rabbit corneal cells by affecting viral immediate-early gene transcription [88].

Three articles reported preclinical pharmacology of marine compounds active against the human immunodeficiency virus type-1 (HIV-1), the causative agent of the acquired immunodeficiency disease syndrome (AIDS), a decrease from our previous review [7]. Ding and colleagues investigated the novel pentacyclic indolosesquiterpene xiamycin (**96**) isolated from the *Streptomyces* sp. GT2002/1503 bacterium and demonstrated selective inhibition against macrophage and T cell β-chemokine receptor CCR5 (5) tropic HIV-infection (estimated IC_50_ = 7.2 μg/mL), with no effect against α-chemokine receptor CXCR4 (X4) tropic HIV [89]. Fan and colleagues isolated several DOPA-derived pyrrole alkaloids, baculiferins I, J, L and M (**97**–**100**), from the Chinese marine sponge *Iotrochota baculifera*, which were found to be potent inhibitors of HIV-1 IIIB (IC_50_ = 0.2–7.0 μM) by binding to the HIV target proteins Vif, APOBEC3G, and gp41 in an as yet undetermined mechanism [90]. Plaza and colleagues isolated several new cyclic depsipeptides from the Indonesian marine sponge *Siliquariaspongia mirabilis* including celebesides A and C (**101**–**102**), which inhibited HIV-1 in an infectivity assay (IC_50_ = 1.9 ± 0.4 μg/mL), thus correlating the anti-HIV activity of these compounds with the presence of phosphoserine [57].

## 3. Marine Compounds with Antidiabetic and Anti-Inflammatory Activity, and Affecting the Immune and Nervous System

Table 2 presents the preclinical pharmacology of marine chemicals (**103**–**162**) which demonstrated antidiabetic and anti-inflammatory activity, as well as affected the immune and nervous system, and whose structures are shown in Figure 2.

**Table 2:** Marine pharmacology in 2009–2011: Marine compounds with antidiabetic and anti-inflammatory activity; and affecting the immune and nervous system.

Drug Class	Compound/organism ^a+^	Chemistry	Pharmacological activity	IC_50_^b^	MMOA ^c^	Country ^d^	References
Antidiabetic	DPHC (**103**)/alga	Polyketide ^e^	Postprandial hyperglycemia inhibition	100 mg/kg *	α-glucosidase and α-amylase inhibition	S. KOR	[104]
Antidiabetic	dysidine (**104**)/sponge	Terpene ^f^	Insulin signaling and glucose uptake	6.7 μM	hPTP1b inhibition	CHN	[105,106]
Anti-inflammatory	arenamides A & B (**105**,**106**)/bacterium	Peptide ^g^	Modulation of LPS-activated murine macrophages *in vitro*	3–10 μM *	Nitric oxide and PGE_2_ inhibition	USA	[107]
Anti-inflammatory	callysterol (**107**)/sponge	Steroid ^f^	Murine hind paw oedema inhibition	ND	TXB_2_ inhibition	EGY, NLD, USA	[108]
Anti-inflammatory	capnellene (**108**)/soft coral	Terpene ^f^	*In vivo* inhibition of microglia activation	10 mg/kg *	iNOS and COX-2 inhibition	TWN	[109]
Anti-inflammatory	elisabethin H (**109**)/soft coral	Terpene ^f^	Modulation of LPS-activated microglia *in vitro*	7.0 μM	TXB_2_ inhibition	USA	[110]
Anti-inflammatory	floridosides (**110**,**111**)/alga	Glycolipid	Free-radical oxidative stress inhibition	22–43 μM *	Myeloperoxidase & MMP inhibition	S.KOR & CHN	[111]
Anti-inflammatory	malyngamide 2 (**112**)/bacterium	PKS/NRPS	LPS-activated macrophage *in vitro* inhibition	8.0 μM	NO inhibition	PNG, USA	[112]
Anti-inflammatory	malyngamide F (**113**)/bacterium	PKS/NRPS	Macrophages NO release & iNOS expression inhibition	7.1 μM	MyD88-dependent pathway inhibition	USA	[113]
Anti-inflammatory	PFF-A (**114**)/alga	Polyketide ^e^	LPS-activated macrophage *in vitro* inhibition	4.7 μM	iNOS and COX-2 inhibition	S. KOR	[114]
Anti-inflammatory	*S. plicata* dermatan sulfate (**115**)/ascidian	Polysaccharide ^h^	Colonic inflammation inhibition	8 mg/kg *	TNF-α, TGF-β, VEGF inhibition	BRA	[115]
Anti-inflammatory	symbiopolyol (**116**)/dinoflagellate	Polyketide ^e^	Lymphocyte adhesion inhibition	6.6 μM	VCAM-1 expression inhibition	JPN	[116]
Anti-inflammatory	tedanol (**117**)/sponge	Terpene ^f^	Murine hind paw oedema inhibition	1 mg/kg *	iNOS, COX-1 and COX-2 inhibition	ITA	[117]
Anti-inflammatory	carijoside A (**118**)/coral	Steroid glycoside ^f^	Neutrophil superoxide and elastase inhibition	1.8–6.8 μg/mL	Undetermined	TWN	[118]
Anti-inflammatory	chabrosterol (**119**)/soft coral	Steroid ^f^	Macrophage COX-2 & iNOS expression inhibition	10 μM *	Undetermined	TWN	[119]
Anti-inflammatory	coscinolactams (**120**–**122**)/sponge	Terpene ^f^	Macrophage PGE_2_ & nitric oxide inhibition	10 μM *	Undetermined	ITA, ESP, FRA	[120]
Anti-inflammatory	durumhemiketalolide C (**123**)/soft coral	Terpene ^f^	Macrophage COX-2 & iNOS expression inhibition	10 μM *	Undetermined	TWN	[121]
Anti-inflammatory	durumolide F (**124**)/soft coral	Terpene ^f^	Macrophage COX-2 & iNOS expression inhibition	10 μM *	Undetermined	TWN	[122]
Anti-inflammatory	gyrosanolides B & C (**125**,**126**)/soft coral	Terpene ^f^	Macrophage iNOS expression inhibition	10 μM *	Undetermined	TWN	[123]
Anti-inflammatory	klysimplexin sulfoxide (**127**) soft coral	Terpene ^f^	Macrophage COX-2 & iNOS expression inhibition	10 μM *	Undetermined	TWN	[124]
Anti-inflammatory	*L. crassum* diterpenes (**128**,**129**)/soft coral	Terpene ^f^	Macrophage NO release & iNOS expression inhibition	3.8–4.0 μM	Undetermined	JPN	[125]
Anti-inflammatory	perthamides C & D (**130**,**131**)/sponge	Peptide^g^	Murine hind paw oedema inhibition	0.3 mg/kg *	Undetermined	FRA, ITA	[126]
Anti-inflammatory	rossinones A & B(**132**,**133**)/ascidian	Terpene ^f^	Neutrophil superoxide inhibition	0.8–2.5 μM	Undetermined	MYS, NZL	[127]
Anti-inflammatory	nebrosteroid I (**134**)/soft coral	Steroid ^f^	Macrophage iNOS expression inhibition	10 μM *	Undetermined	TWN	[128]
Anti-inflammatory	sarcoehrenosides A & B (**135**,**136**)/soft coral	Glycolipid	Macrophage iNOS expression inhibition	10 μM *	Undetermined	TWN	[129]
Anti-inflammatory	sarcocrassocolides A & B(**137**,**138**)/soft coral	Terpene ^f^	Macrophage iNOS expression inhibition	10 μM *	Undetermined	TWN	[130]
Anti-inflammatory	simplexin E (**139**)/soft coral	Terpene ^f^	Macrophage COX-2 & iNOS expression inhibition	10 μM *	Undetermined	TWN	[131]
Anti-inflammatory	terpioside B (**140**)/sponge	Glycolipid	Macrophage iNOS expression inhibition	<10 μM *	Undetermined	ITA	[132]
Immune system	grassystatins A–C (**141**–**143**)/bacterium	Peptide ^g^	T cell antigen presentation inhibition	10 μM *	Cathepsin E, IL-17 and IFN-γ inhibition	USA	[133]
Immune system	callyspongidiol (**144**) & 14,15-dihydrosiphonodiol (**145**)/sponge	Polyketide ^e^	Dendritic cell activation	10 μM *	IL-10 and Ag-presenting activity	DEU, JPN	[134]
Immune system	PFF-A (**114**)/alga	Polyketide ^e^	Basophil IgE receptor inhibition	25 μM *	Ca^2+^ influx and degranulation inhibition	S. KOR	[135]
Immune system	splenocin B (**146**)/bacterium	PKS/NRPS	Interleukin 5 and 13 Inhibition	1.6–1.8 nM	Undetermined	USA	[136]
Immune system	HCLPS-1 (**147**)/clam	Polysaccharide ^h^	*In vivo* & *in vitro* T and B cell activation	20 mg/kg *	Undetermined	CHN	[137]
Immune system	yessotoxin (**148**)/alga	Polyketide(polyether) ^e^	Macrophage phagocytosis inhibition	1 nM *	TNF-α, MIP-1α & MIP-2 inhibition	ITA	[138]
Nervous system	calyculin A (**149**)/sponge	PKS/NRPS ^e^	Hippocampal neuron neurite retraction	100 mM *	Dependent on actomyosin activation	JPN	[139]
Nervous system	*C. olemda* purine (**150**)/sponge	Alkaloid ^g^	Convulsion induction	4 nm/mouse *	GABAergic transmission inhibition	JPN, USA	[140]
Nervous system	hoiamide B (**151**)/bacterium	Peptide ^g^	Neocortical neuron Ca^2+^ oscillation inhibition	79.8 nM	Stimulation of sodium influx	ITA, PNG, USA	[141]
Nervous system	palmyrolide A (**152**)/bacterium	Polyketide ^e^	Neocortical neuron Ca^2+^ oscillation inhibition	3.7 µM	Sodium influx inhibition	MEX, USA	[142]
Nervous system	xyloketal B (**153**)/fungus	Polyketide ^e^	Ischemia-induced PC12 cell injury inhibition	100 µM *	Free radical scavenging	CHN	[143]
Nervous system	alotamide (**154**)/bacterium	PKS/NRPS ^g^	Neocortical neuron Ca^2+^ oscillation stimulation	4.18 μM	Undetermined	MEX, USA	[144]
Nervous system	(−)-dibromophakellin (**155**)/sponge	Alkaloid ^g^	Α_2B_ adrenoreceptor agonist	4.2 µM	Undetermined	AUS	[145]
Nervous system	dysideamine (**156**)/sponge	Terpene ^e^	Hippocampal reactive oxygen species inhibition	10 µM *	Undetermined	IDN, JPN	[146]
Nervous system	ircinialactams (**157**,**158**)/sponge	Terpene ^f^	α1 & α3 glycine receptor potentiation	0.5 µM *	Undetermined	AUS	[147]
Nervous system	eusynstyelamides B & C (**159**,**160**)/ascidian	Peptide ^g^	Neuronal nitric oxide synthase inhibition	4.3–5.8 µM	Undetermined	AUS	[148]
Nervous system	nanolobatolide (**161**)/soft coral	Terpene ^f^	6-hydroxy-dopamine neurotoxicity inhibition	0.1 µM *	Undetermined	TWN	[149]
Nervous system	pulicatin A (**162**)/bacterium	Alkaloid ^g^	Human serotonin 5-HT_2B_ binding	505 nM **	Undetermined	PHL, USA	[150]

^a^ **Organism:** *Kingdom Animalia*: ascidian (Phylum Chordata), coral (Phylum Cnidaria), clam (Phylum Mollusca), fireworm (Phylum Polychaeta), sponge (Phylum Porifera); *Kingdom Chromalveolata*: dinoflagellates (Phylum Dinoflagellata); *Kingdom Fungi*: fungus; *Kingdom Plantae*: alga; *Kingdom Monera*: bacterium; ^b^ **IC_50_**: concentration of a compound required for 50% inhibition, *: apparent IC_50_; **: Ki; ND: not determined; ^c^ **MMOA**: molecular mechanism of action, NO: nitric oxide; ^d^ **Country**: AUS: Australia; BRA: Brazil; CHN: China; DEU: Germany; EGY: Egypt; ESP: Spain; FRA: France; IDN: Indonesia; ITA: Italy; JPN: Japan; MEX: Mexico; MYS: Malaysia; NLD: The Netherlands; NZL: New Zealand; PNG: Papua New Guinea; PHL: Phillipines; S.KOR: South Korea; TWN: Taiwan; ^e^ **Chemistry**: Polyketide; ^f^ Terpene; ^g^ Nitrogen-containing compound; ^h^ polysaccharide, modified as in the text.

### 3.1. Antidiabetic Activity

Heo and colleagues extended the pharmacology of diphlorethohydroxycarmalol [DPHC] (**103**), previously isolated from the marine brown alga *Ishige okamurae*, by showing that DPHC alleviated postprandial hyperglycemia in diabetic mice by potent inhibition of both α-glucosidase and α-amylase enzymes (IC_50_ = 0.16 and 0.53 nM, respectively) suggesting a possible use of DPHC “as a nutraceutical or functional food for diabetes” [104]. Li and colleagues evaluated the known sesquiterpene dysidine (**104**) from the Hainan marine sponge *Dysidea villosa*, and revealed that it activated the insulin pathway by inhibition of human protein phosphatase 1B (IC_50_ = 6.70 µM), a well characterized drug target for type-II diabetes and obesity treatment, as well as glucose uptake and glucose transporter 4 translocation *in vitro* [105,106].

### 3.2. Anti-Inflammatory Activity

There was a remarkable increase in marine anti-inflammatory pharmacology research during 2009–2011. The molecular mechanism of action of several marine natural products, which were shown in preclinical pharmacological studies to target neutrophils and macrophages both *in vitro* and *in vivo*, was reported in several publications. Asolkar and colleagues described two new cyclohexadepsipeptides arenamides A and B (**105**,**106**), isolated from the Fijian bacterium *Salinispora arenicola*, that inhibited LPS-induced murine macrophage RAW 264.7 cells PGE_2_ and NO production *in vitro*, by affecting NFκB signaling activity (IC_50_ = 3.7 and 1.7 μM, respectively), thus highlighting their “anti-inflammatory characteristics” [107]. Three publications yielded potentially novel compounds targeting proinflammatory mediators released by activated brain microglia, a macrophage involved in neuroinflammation and neurodegeneration [151]: Youssef and colleagues described a new steroid callysterol (**107**) from the Red Sea sponge *Callyspongia siphonella*, which potently inhibited rat hind paw edema with an activity close to cortisone, and also reduced TXB_2_ release from LPS-activated rat brain microglia (apparent IC_50_ > 10 μM) [108]. Jean and colleagues observed that the sesquiterpene capnellene (**108**) isolated from the Indonesian soft coral *Capnella imbricate*, attenuated expression of inducible cyclooxygenase-2 both in activated microglia *in vitro* and *in vivo*, suggesting it might contribute to “the search for new therapeutic agents for treatment of neuroinflammatory diseases” [109]. Shi and colleagues isolated a new terpene, elisabethin H (**109**) from the Caribbean gorgonian octocoral *Pseudopterogorgia elisabethae*, which significantly inhibited superoxide anion (O_2_^–^) generation from *E. coli* LPS activated rat neonatal microglia *in vitro* (IC_50_ = 7 μM) [110]. Li and colleagues reported that the floridosides (**110**,**111**), isolated from the South Korean marine red alga *Laurencia undulata*, possessed significant antioxidant capacity and inhibited the proinflammatory matrix metalloproteinases MMP-2 and MMP-9, thus suggesting they might be candidates for further development as natural marine antioxidants [111]. Three publications investigated inhibition of pro-inflammatory mediators released by activated macrophage cell lines: Malloy and colleagues reported that the lipopeptide malyngamide 2 (**112**) from the Papua New Guinea marine cyanobacterium *Lyngbya sordida* inhibited nitric oxide production in LPS-primed RAW 264.7 macrophage cells (IC_50_ = 8.0 μM) [112]. In a detailed mechanistic study Villa and colleagues investigated the lipopeptide malyngamide F (**113**) from the marine cyanobacterium *Lyngbya majuscula* showing that it inhibited nitric oxide production in LPS-primed RAW 264.7 macrophage cells (IC_50_ = 7.1 μM) by selectively inhibiting the MyD88-dependent pathway of TLR4 and 9, thus potentially becoming a “useful tool” in cellular biology [113]. Kim and colleagues extended previous studies with the phlorotannin phlorofucofuroeckol A (PFF-A) (**114**), isolated from the Korean brown alga *Ecklonia stolonifera*, by demonstrating its antioxidant activity was of similar potency to vitamin C, and that it inhibited nitric oxide and PGE_2_ production (apparent IC_50_ = 5–10 μM) by downregulation of iNOS and COX-2 protein expression in LPS-primed RAW 264.7 macrophage cells [114]. Using an *in vivo* rat colitis model, Belmiro and colleagues provided a detailed molecular characterization of the anti-inflammatory properties of a dermatan sulfate (**115**), analog of mammalian heparin, purified from the Brazilian ascidian *Styela plicata*, which at 8 mg/kg per day significantly decreased lymphocyte and macrophage recruitment as well as TNF-α, TGF-β, and VEGF production in the inflamed rat colon [115]. Hanif and colleagues reported that the highly hydroxylated long-chain sulfate symbiopolyol (**116**), isolated from a symbiotic dinoflagellate of the jellyfish *Mastigias papua* significantly inhibited (K_50_ = 6.6 μM) the expression of the inducible adhesion vascular cell adhesion molecule-1 which binds to leukocytes present in early stages of inflammation, and thus might become a “potential anti-inflammatory agent” [116]. Costantino and colleagues reported that tedanol (**117**), a new brominated and sulfated pimarane diterpene isolated from the Caribbean sponge *Tedania ignis*, significantly reduced both the acute and subchronic phases of carrageenan-induced inflammation at 1 mg/kg with concomitant inhibition of both COX-2, iNOS expression and cellular infiltration [117].

As shown in Table 2 and in contrast to the marine anti-inflammatory compounds previously discussed, while an anti-inflammatory activity and IC_50_ were reported, the molecular mechanism of action remained undetermined for the following marine compounds: carijoside A (**118**) [118]; chabrosterol (**119**) [119]; coscinolactams (**120**–**122**) [120]; durumhemiketalolide C (**123**) [121]; durumolide F (**124**) [122]; gyrosanolides B and C (**125**,**126**) [123]; klysimplexin sulfoxide C (**127**) [124]; *L. crassum* diterpenes (**128**,**129**) [125]; perthamides C and D (**130**,**131**) [126]; rossinones A and B (**132**,**133**) [127]; nebrosteroid I (**134**) [128]; sarcoehrenosides A and B (**135**,**136**) [129]; sarcocrassocolides A & B (**137**,**138**) [130]; simplexin E (**139**) [131]; and terpioside B (**140**) [132].

### 3.3. Marine Compounds with Activity on the Immune System

In 2009–2011, immune system pharmacology of marine compounds showed a considerable decrease from our previous review.

Kwan and colleagues isolated the linear peptides grassystatins A–C (**141**–**143**) from a marine cyanobacterium identified as *Lyngbya* cf. *confervoides*, demonstrating that the compounds selectively inhibited aspartic protease cathepsin E (IC_50_ = 0.3–0.8 nM) *versus* cathepsin D, as well as antigen-stimulated T cell proliferation, with a concomitant reduction of IL-17 and interferon γ [133]. Takei and colleagues reported that the known compounds callyspongidiol (**144**) and 14,15-dihydrosiphonodiol (**145**), isolated from a marine sponge *Callyspongia* sp., activated both functional and phenotypic maturation of human monocyte-derived dendritic cells, as well as higher interleukin-10 production by T cells, thus revealing potential use in autoimmune diseases and cancer [134]. Shim and colleagues observed that the phloroglucinol derivative phlorofucofuroeckol A (PFF-A) (**114**), purified from the Korean marine seaweed *Ecklonia stolonifera*, reduced the expression of the human basophil FcεR1 receptor (apparent IC_50_ = 25 µM), as well as intracellular [Ca^2+^]_i_ and histamine release, findings which may be relevant for regulation of IgE-mediated allergic reactions [135]. Strangman and colleagues discovered novel splenocin B (**146**) isolated from the marine bacterium *Streptomyces* species strain CNQ431 that displayed potent inhibition of murine splenocyte-derived T_H_2 cytokines interleukin 5 and 13 (IC_50_ = 1.6–1.8 nM), thus contributing to the development of a “splenocin-derived drug” for allergic inflammation [136]. Dai and colleagues isolated a water soluble polysaccharide HCLPS-1 (**147**) from the Chinese pearl-producing mollusc *Hyriopsis cumingii* Lea, which stimulated murine spleen lymphocyte proliferation *in vitro* and *in vivo* in a concentration-dependent manner (apparent IC_50_ less than 20 mg/kg), suggesting HCLPS-1 might become a “potential natural immunomodulator” upon further pharmacological study [137]. Orsi and colleagues contributed to the immunopharmacology of the sulfated dinoflagellate polyether yessotoxin (**148**), by demonstrating that it decreased macrophage phagocytic activity against the fungus *Candida albicans* (apparent IC_50_ = 1 nM), affected the cytoskeleton by inducing F-actin re-organization, and enhanced release of the cytokine TNF-α and chemokines MIP-1α and MIP-2 [138].

### 3.4. Marine Compounds Affecting the Nervous System

As shown in Table 2, the nervous system pharmacology of marine natural products in 2009–2011 involved some areas of neuropharmacology, namely neuronal neurite retraction, neurotransmission inhibition, neuronal Ca^2+^ oscillations and free radical inhibition.

Marine natural products have previously been reported to affect neuritogenesis [7], a process required by neurons to respond to the extracellular environment to form synaptic connections. Inutsuka and colleagues contributed novel molecular studies on the effect of calyculin A (**149**) on neurons, demonstrating that rapid rat hippocampal neuron neurite retraction (apparent IC_50_ = 100 mM) induced by the toxin was dependent on actin filament polymerization or myosin II motor, yet independent of the microtubule polymerization status, and perhaps resulted from dephosphorylation of myosin light chain kinase [139]. Sakurada and colleagues reported that the novel Palauan sponge *Cribrochalina olemda* purine (**150**), which elicited convulsions upon intracerebroventricular injections in mice (4 nM/mouse), inhibited GABAergic transmission in hippocampal neurons [140]. Noteworthy was the author’s observation that this marine purine was “closely related in structure to endogenous neurosignaling molecules and commonly used CNS stimulants”.

As shown in Table 2, two marine compounds (**151**,**152**) identified as part of a drug discovery screening program, were shown to inhibit neuronal Ca^2+^ oscillations, a network phenomenon that appears to depend on voltage-gated sodium channel activation. Choi and colleagues reported that a cyclic depsipeptide hoiamide B (**151**), isolated from a Papua New Guinean cyanobacteria assemblage of *Symploca* sp. and *Oscillatoria* cf. sp., stimulated sodium influx (IC_50_ = 3.9 µM) in murine neuronal cells *in vitro* by putative activation of site 2 on the sodium channel, while suppressing spontaneous Ca^2+^ (IC_50_ = 79.8 nM) with greater potency, thus revealing that hoiamide B may have more than one molecular target [141]. Pereira and colleagues contributed a novel marine macrolide palmyrolide A (**152**) isolated from Northern Pacific Palmyra Atoll cyanobacteria assemblages of *Leptolyngbya* cf. and *Oscillatoria* sp. that inhibited both sodium influx (IC_50_ = 5.2 µM) in mouse neuroblastoma cells and spontaneous Ca^2+^ oscillations (IC_50_ = 3.7 µM) in primary cultures of murine cerebrocortical neurons, “making it an intriguing candidate for further pharmacological exploration” [142]. Zhao and colleagues reported that the known compound xyloketal B (**153**), isolated from the marine mangrove fungus *Xylaria* sp., inhibited ischemia-induced PC12 cell injury (IC_50_ = 100 µM) by a neuroprotective mechanism involving free radical scavenging and reduction of mitochondrial membrane potential and superoxide generation, suggesting further development for effective stroke therapy [143].

Finally, and as presented in Table 2, during 2009–2011, several other marine compounds were reported to affect the nervous system by demonstrating pharmacological activity on Ca^2+^ oscillations, several receptors, and neuronal nitric oxide synthase, yet the mechanism of action of these compounds remained undetermined: alotamide A (**154**) [144], (−)-dibromophakellin (**155**) [145], dysideamine (**156**) [146], ircinialactams (**157**,**158**) [147], eusynstyelamides B and C (**159**,**160**) [148], nanolobatolide (**161**) [149], and pulicatin A (**162**) [150].

## 4. Marine Compounds with Miscellaneous Mechanisms of Action

Table 3 presents the 2009–2011 preclinical pharmacology of 68 marine compounds (**163**–**226**) with miscellaneous mechanisms of action, with their respective structures shown in Figure 3. Because additional *in vitro* and *in vivo* pharmacological data for these compounds remained unpublished, assignment of these marine compounds to a particular drug class was not possible.

As shown in Table 3, the peer-reviewed literature reported a pharmacological activity, an IC_50_, and a molecular mechanism of action for 21 marine natural products: bisebromoamide (**163**) [152]; botryllamides I and J (**164**,**165**) [153]; dysidine (**104**) [106]; *Ecklonia cava* phlorotannins (**166**,**167**) [154]; fucophlorethols (**168**,**169**) [155]; gonad-stimulating substance (GSS) (**170**) [156]; halichlorine (**171**) [157]; hoiamide A (**172**) [158]; hypochromins A and B (**173**,**174**) [159]; mycothiazole (**175**) [160]; *Mycale* sp. pyrroles (**176**,**177**) [161]; neocomplanines A and B (**178**,**179**) [162]; pateamine A (**180**) [163]; polytheonamide B (**181**) [164]; and zampanolide (**182**) [165].

In contrast, although a pharmacological activity was described, and an IC_50_ for inhibition of an enzyme or receptor determined, detailed molecular mechanism of action studies were unavailable at the time of publication for the following 47 marine compounds included in Table 3: alotaketals A and B (**183**,**184**) [166]; aquastatin A (**185**) [167]; australin E (**186**) [168]; lyngbyastatins 9 & 10 (**187**,**188**) [169]; brunsvicamides A, B and C (**189**–**191**) [170]; carteriosulfonic acids A, B and C (**192**–**194**) [171]; *Carteriospongia foliascens* sesterterpenoids (**195**,**196**) [172]; clavatadines D and E (**197**,**198**) [173]; fibrosterol sulfates A and B (**199**,**200**) [174]; gracilin L (**201**) [175]; grassystatins A, B and C (**141**–**143**) [133]; 2-hydroxycircumdatin C (**202**) [176]; jaspaquinol (**203**) [177]; largamides A, B and C (**204**–**206**) [178]; largamide D oxazolidine (**207**) [179]; *Laurencia similis* brominated metabolites (**208**,**209**) [180]; molassamide (**210**) [181]; myrothenone A (**211**) [182]; 42-hydroxy-palytoxin (**212**) [183]; plectosphaeroic acids A, B and C (**213**–**215**) [184]; puupehenone (**216**) [177]; *Sinularia numerosa* oxylipin (**217**) [185]; sipholenone E (**218**) [186]; spartinoxide (**219**) [187]; 23-*nor*-spiculoic acid B (**220**) [188]; tanikolide dimer (**221**) [189]; tamulamides A and B (**222**,**223**) [190]; terretonins E and F (**224**,**225**) [191]; and tetrangulol methyl ether (**226**) [192].

**Table 3:** Marine pharmacology in 2009–2011: Marine compounds with miscellaneous mechanisms of action.

Compound/Organism ^a^	Chemistry	Pharmacological Activity	IC_50_^b^	MMOA ^c^	Country ^d^	References
bisebromoamide (**163**)/cyanobacterium	Peptide ^g^	*In vitro* tumor growth inhibition	0.040 μM	ERK inhibition	JPN	[152]
botryllamide I & J (**164**,**165**)/ascidian	Shikimate ^g^	Multidrug resistance inhibition	27–41 μM	ABCG2 transporter inhibition	USA	[153]
dysidine (**104**)/sponge	Terpene ^f^	Insulin pathway activation	10 μM	Protein tyrosine phosphatase 1B inhibition	CHN	[106]
*E. cava* phlorotannins (**166**,**167**)/alga	Polyketide ^e^	*In vitro* antioxidants		DPPH, hydroxyl, peroxyl, & superoxide scavenging	CHN, S. KOR	[154]
fucophlorethols (**168**,**169**)/alga	Polyketide ^e^	DPPH radical scavenging	10–14 μM	Cytochrome P450 CYP1A inhibition	DEU, ISR	[155]
GSS (**170**)/starfish	Peptide ^g^	Oocyte maturation and ovulation	2 nM	cAMP production	JPN	[156]
halichlorine (**171**)/sponge	Alkaloid (polyketide) ^g^	Inhibition of vascular contractility	3 μM *	L-type Ca^2+^ channel inhibition	JPN	[157]
hoiamide A (**172**)/bacterium	Peptide ^g^	Voltage-gated sodium channel activator	2.3 μM	Sodium channel site 2 activator	USA	[158]
hypochromin A & B (**173**,**174**)/fungus	Polyketide ^e^	Angiogenesis inhibition	13 & 50 μM	Tyrosine kinase inhibition	JPN	[159]
mycothiazole (**175**)/sponge	PKS/NRPS	Angiogenesis inhibition	10 nM *	Mitochondrial complex 1 inhibition	USA	[160]
*Mycale* sp. metabolites (**176**,**177**)/sponge	Polyketide ^e^	Hypoxia-inducible factor-1 inhibition	7.8–8.6 μM	Mitochondrial electron transport chain inhibition	USA	[161]
neocomplanines A & B (**178**,**179**)/fireworm	Polyketide ^e^	Murine footpad inflammation	ND	PKC activation	JPN	[162]
pateamine A (**180**)/sponge	PKS/NRPS	Nonsense-mediated mRNA inhibition	100 nM *	Binding to eukaryotic initiation factor 4AIII	DEU, USA	[163]
polytheonamide B (**181**)/sponge	Peptide ^g^	Cytotoxic mammalian channel formation	14–29 nM	Selectivity towards Cs + cation	JPN	[164]
zampanolide (**182**)/sponge	Polyketide ^e^	G2/M cell cycle arrest	8 nM *	Microtubule bundle formation by tubulin polymerization	NZL	[165]
alotaketals A & B (**183**,**184**)/sponge	Terpene ^f^	cAMP cell signaling activation	18 & 240 nM	Undetermined	CAN, NLD, PAP	[166]
aquastatin (**185**)/fungus	Polyketide ^e^	Protein phosphatase 1B inhibition	0.19 μM	Undetermined	S. KOR	[167]
australin E (**186**)/soft coral	Terpene ^f^	Inositol 5-phosphatase SHIP1 activation	>100 μM	Undetermined	CAN	[168]
lyngbyastatins 9 & 10 (**187**,**188**)/bacterium	Peptide ^g^	Elastase and chymotrypsin inhibition	0.2–9.3 μM	Undetermined	USA	[169] *
brunsvicamides A–C (**189**–**191**)/bacterium	Peptide ^g^	Elastase inhibition	2.0–4.4 μM	Undetermined	DEU	[170]
carteriosulfonic acids A, B & C (**192**–**194**)/sponge	Polyketide ^e^	GSK-3β inhibition	6.8–12.5 µM	Undetermined	SGP, USA	[171]
*Carteriospongia foliascens* sesterterpenoids (**195**,**196**)/sponge	Terpene ^f^	Human Ras-converting enzyme inhibition	4.2 μg/mL *	Undetermined	CAN, IDN,NLD, USA	[172]
clavatadines D & E (**197**,**198**)/sponge	Shikimate ^g^	Factor XIa inhibition	222 μM *	Undetermined	AUS	[173]
fibrosterol sulfates A & B (**199**,**200**)/sponge	Terpene ^f^	Protein Kinase Cζ inhibition	5.6 & 16.4 µM	Undetermined	PHL, USA	[174]
gracilin L (**201**)/sponge	Terpene ^f^	EGF-R tyrosine kinase inhibition	<100 μM *	Undetermined	GBR, LUX	[175]
grassystatins A–C (**141**–**143**)/bacterium	Peptide ^g^	cathepsin E inhibition	0.3–43 nM	Undetermined	USA	[133]
2-hydroxycircumdatin C (**202**)/fungus	Alkaloid ^g^	DPPH radical scavenging activity	9.9 µM	Undetermined	CHN	[176]
jaspaquinol (**203**)/sponge	Terpene ^f^	5-lipoxygenase inhibition	0.45 µM	Undetermined	USA	[177]
largamides A–C (**204**–**206**)/bacterium	Peptide ^g^	Elastase inhibition	0.53–1.41 µM	Undetermined	USA	[178]
largamide D oxazolidine (**207**)/bacterium	Peptide ^g^	Elastase and chymotrypsin inhibition	0.9–1.5 μM	Undetermined	USA	[179]
*Laurencia similis* brominated metabolites (**208**,**209**)/alga	Polyketide ^e^	Protein phosphatase 1B inhibition	2.7–3 μM	Undetermined	CAN, CHN	[180]
molassamide (**210**)/bacterium	Peptide ^g^	Elastase and chymotrypsin inhibition	0.03 & 0.23 μM	Undetermined	USA	[181]
myrothenone A (**211**)/fungus	Polyketide ^e^	Tyrosinase inhibition	6.6 μM	Undetermined	S. KOR	[182]
42-hydroxy-palytoxin (**212**)/soft coral	PKS/NRPS	Na^+^/K^+^ pump inhibition	28 ± 7 nM	Undetermined	ITA, USA	[183]
plectosphaeroic acids A–C (**213**–**215**)/fungus	Alkaloid ^g^	Indoleamine 2, 3 dioxygenase inhibtion	2 μM *	Undetermined	CAN	[184]
puupehenone (**216**)/sponge	Terpene ^f^	5-lipoxygenase inhibition	0.68 μM	Undetermined	USA	[177]
*Sinularia numerosa* oxylipin (**217**)/soft coral	Fatty acid ^e^	Angiogenesis inhibition	20–40 μM	Undetermined	JPN	[185]
sipholenone E (**218**)/sponge	Terpene ^f^	P-glycoprotein multidrug resistance reversal	5.7–62 nM	Undetermined	EGY, CHN, USA	[186]
spartinoxide (**219**)/fungus	Terpene ^f^	Human elastase inhibition	6.5 μM	Undetermined	DEU	[187]
23-*nor*-spiculoic acid B (**220**)/sponge	Polyketide ^e^	NFκB inhibition	0.47 μM	Undetermined	VEN, USA	[188]
tanikolide dimer (**221**)/bacterium	Polyketide ^e^	Human sirtuin type 2 inhibition	0.176–2.4 μM	Undetermined	DEU, S. KOR, USA	[189]
tamulamide A & B (**222**,**223**)/dinoflagellate	Polyketide(polyether) ^e^	Brevetoxin-3 binding inhibition	0.2–2.5 μM	Undetermined	USA	[190]
terretonins E & F (**224**,**225**)/fungus	Terpene ^f^	NADH oxidase inhibition	2.9–3.9 μM	Undetermined	ESP, ITA	[191]
tetrangulol methyl ether (**226**)/bacterium	Polyketide ^e^	Quinone reductase-2 inhibition	0.16 μM	Undetermined	USA	[192]

^a^ **Organism**, *Kingdom Animalia*: ascidian (Phylum Chordata), fireworm (Phylum Annelida), soft corals (Phylum Cnidaria), starfish ( Phylum Echinodermata), sponge (Phylum Porifera); *Kingdom Chromalveolata*: dinoflagellates; *Kingdom Fungi*: fungus; *Kingdom Plantae*: alga; *Kingdom Monera*: bacterium; ^b^ **IC_50_**: concentration of a compound required for 50% inhibition *in vitro*; *: estimated IC_50_; ^c^ **MMOA**: molecular mechanism of action; ^d^ **Country:** AUS: Australia; CAN: Canada; CHN: China; DEU: Germany; EGY: Egypt; ESP: Spain; GBR: United Kingdom; IDN: Indonesia; ISR: Israel; ITA: Italy; JPN: Japan; LUX: Luxembourg; NZL: New Zealand; NLD: The Netherlands; PHL: Phillipines; PAP: Papua New Guinea; SGP: Singapore; S. KOR: South Korea; ESP: Spain; VEN: Venezuela; ^e^ **Chemistry:** Polyketide; ^f^ Terpene; ^g^ Nitrogen-containing compound; *: Bouillamides A and B are identical with lyngbyastatins 9 and 10. See [193].

## 5. Reviews on Marine Pharmacology

Several reviews covering both general and specific areas of marine preclinical pharmacology were published during 2009–2011: (a) ***marine pharmacology and marine pharmaceuticals***: a renaissance in marine pharmacology: from preclinical curiosity to clinical reality [194]; biologically active marine natural products [195]; drug development from marine natural products [196]; biotechnological potential of marine natural products [197]; pharmaceuticals from marine natural products: surge or ebb? [198]; marine pharmacology in Australia: the Roche Research Institute [199]; the global marine pharmaceutical pipeline in 2010: U.S. Food and Drug Administration-approved compounds and those in Phase I, II and III of clinical development [200]; marine drugs from sponge-microbe associations [201]; cyanobacteria as an emerging source for drug discovery [202]; marine invertebrates as a future therapeutic treasure [203]; biodiversity conservation and marine natural products drug discovery [204]; marine invertebrates as a source of guanidines with chemical and pharmacological significance [205]; innovations in the field of marine natural products and a new wave of drugs [206]; (b) ***antimicrobial marine pharmacology***: antibacterial marine natural products [207]; marine microbes and pharmaceutical development [208]; marine microbe-derived antibacterial agents [209]; antimicrobial peptides from marine invertebrates [210]; novel anti-infective compounds from marine bacteria [211]; conventional and unconventional antimicrobials from fish, marine invertebrates and microalgae [212]; (c) ***antiviral marine pharmacology***: antiviral lead compounds from marine sponges [213]; potential anti-HIV agents from marine resources [214]; marine compounds and their antiviral activities [215]; marine organisms as a therapeutic source against herpes simplex virus infection [216]; (d) ***antiparasitic,antituberculosis, antimalarial and antifungal marine pharmacology***: antiparasitic marine invertebrate-derived small molecules [217]; marine antileishmanial natural products [218]; antituberculosis leads from marine microbial metabolites [219]; antimalarial drug discovery from marine sources between January 2003 and December 2008 [220]; antimalarial marine natural products from 2006 to 2008 [221]; antimalarial marine compounds [222]; (e) ***immuno- and anti-inflammatory marine pharmacology***: marine natural product leads for treatment of inflammation [223]; marine natural products targeting phospholipase A_2_ [224]; marine diterpene glycosides as anti-inflammatory agents [225]; anti-inflammatory compounds from marine algae [226]; (f) ***cardiovascular marine pharmacology***: marine-derived angiotensin-I-converting enzyme inhibitors [227]; (g) ***nervous system marine pharmacology***: conotoxins as natural products drug leads [228]; marine indole alkaloids as new drug leads for depression and anxiety [229]; marine natural products and ion channel pharmacology [230]; neuroprotective effects of marine algae [231]; conopeptides as novel options for pain management [232]; structure-activity studies with α-conotoxins as selective antagonists of nicotinic acetylcholine receptors [233]; (h) ***miscellaneous molecular targets***: calyculins and related marine natural products as serine threonine protein phosphatase inhibitors [234]; NF-κB inhibition by marine natural products [235]; protein kinase inhibitors from marine sponges [236].

## 6. Conclusions

The global marine preclinical and clinical pharmaceutical pipelines remain remarkably active one year after U.S. Food and Drug Administration approval of brentuximab vedotin (Adcetris^®^), a conjugate between a monoclonal antibody that targets the cell-membrane protein CD30, an antigen which is highly expressed in lymphoid tumors, and several units of the potent antimitotic agent monomethyl auristatin E, a synthetic analog of the marine compound dolastatin 10 [237].

This review aims to continue contributing to the marine *preclinical* pipeline review series that was initiated in 1998 [1,2,3,4,5,6,7] and reveals the breadth of preclinical pharmacological research during 2009–2011, resulting from the global research effort of chemists and pharmacologists from Australia, Belgium, Brazil, Canada, China, Colombia, Cuba, Egypt, Fiji, France, Germany, Indonesia, Israel, Italy, Japan, Luxemburg, Malaysia, Mexico, the Netherlands, New Caledonia, New Zealand, Norway, Panama, Papua New Guinea, Philippines, South Africa, South Korea, Singapore, Spain, Switzerland, Taiwan, Thailand, United Kingdom, Venezuela, Vietnam, and the United States. Thus, we feel confident to predict that the marine *preclinical* pharmaceutical pipeline will most probably continue to provide novel pharmacological lead compounds that will enrich the marine *clinical* pharmaceutical pipeline [200], which currently consists of 6 U.S. Food and Drug Administration-approved pharmaceuticals and 11 compounds in Phase I, II and III of clinical development and which may be viewed at [238].
